# *Trans*-translation exposed: understanding the structures and functions of tmRNA-SmpB

**DOI:** 10.3389/fmicb.2014.00113

**Published:** 2014-03-21

**Authors:** Emmanuel Giudice, Kevin Macé, Reynald Gillet

**Affiliations:** ^1^Translation and Folding Team, Université de Rennes 1, CNRS UMR 6290 IGDRRennes, France; ^2^Institut Universitaire de FranceFrance

**Keywords:** ribosome, tmRNA, SmpB, *trans*-translation, structure

## Abstract

Ribosome stalling is a serious issue for cell survival. In bacteria, the primary rescue system is *trans*-translation, performed by tmRNA and its protein partner small protein B (SmpB). Since its discovery almost 20 years ago, biochemical, genetic, and structural studies have paved the way to a better understanding of how this sophisticated process takes place at the cellular and molecular levels. Here we describe the molecular details of *trans*-translation, with special mention of recent cryo-electron microscopy and crystal structures that have helped explain how the huge tmRNA-SmpB complex targets and delivers stalled ribosomes without interfering with canonical translation.

## Introduction

**Figure 1:**
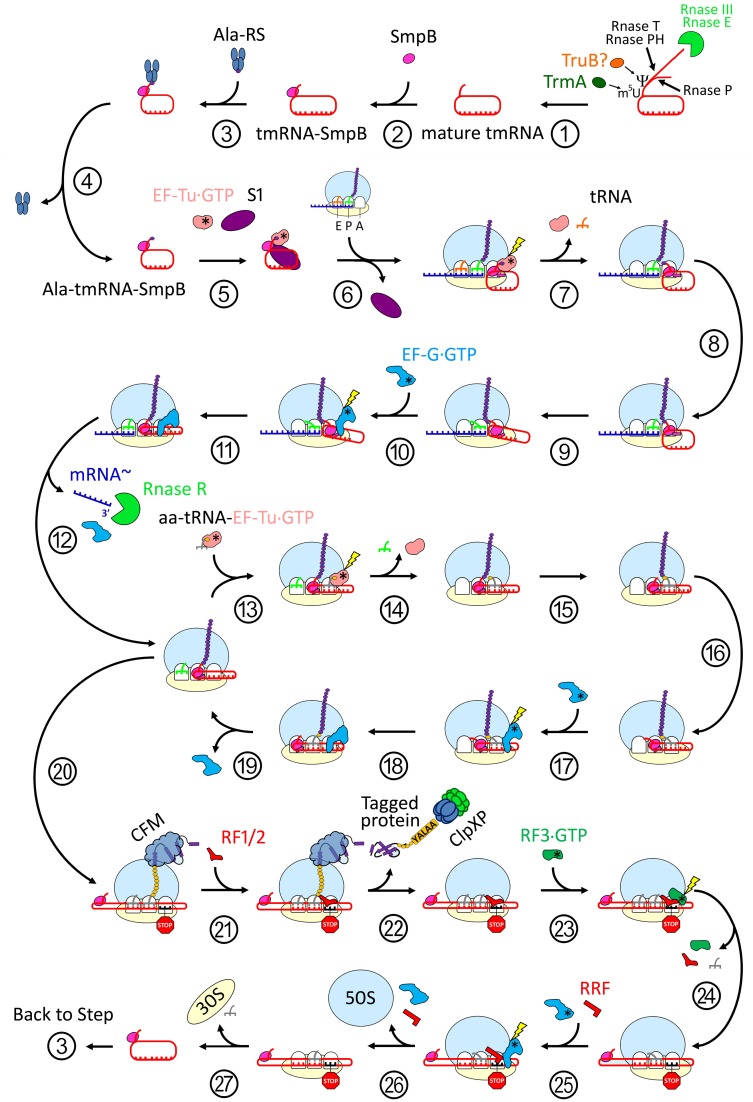
**The cycle of *trans*-translation**. Maturation: (1) The tmRNA primary transcript 5′-terminus is processed by the endonuclease RNAse P, while the 3′-terminus is first cleaved by endonucleases RNAses III or E then trimmed by exonucleases RNAses T and/or PH. Nucleotides in the T-loop are modified at least twice: a 5-methyluridine is catalysed by TrmA, and a pseudouridine may be catalyzed by TruB. (2) The tmRNA-SmpB complex is formed. (3) Ala-RS charges the deacyl tmRNA-SmpB with a new alanine. (4) Ala-RS is released. (5) EF-Tu•GDP and S1 bind to Ala-tmRNA^Ala^-SmpB, and the complex is ready to rescue a stalled ribosome. Re-registration: (6) Pre-accommodation. The ala-tmRNA^Ala^-SmpB-EF-Tu•GTP quaternary complex binds to a stalled ribosome. SmpB recognizes the vacant A-site. S1 is released. (7) SmpB simulates the codon-anticodon recognition and induces GTPase activity on EF-Tu. Ala-tmRNA^Ala^-SmpB accommodates into the A-Site. EF-Tu•GDP and E-Site deacyl tRNA are released. (8) Peptidyl transfer. The nascent peptide is transferred from the P-Site tRNA to the Ala-tmRNA^Ala^. The nascent peptide is elongated by one Ala. (9) Ratchet. The 30S subunit spontaneously rotates in an anticlockwise direction relative to the 50S. This ratchet-like motion brings TLD-SmpB and tRNA into hybrid states of binding (A/P and P/E respectively). (10) EF-G•GTP binds to the ribosome, stabilizing the ratchet formation and inducing a unique 12° head tilt. (11) GTP hydrolysis. TLD-SmpB and tRNA are translocated to the P- and E-sites, respectively. The tmRNA internal ORF is positioned in the A-site. (12) EF-G•GDP and non-stop mRNA release. Subsequent degradation of non-stop mRNA by RNAse R. Elongation: translation restart on the tmRNA internal ORF: (13) aa-tRNA^*aa*^-EF-Tu•GDP ternary complex binds to the ribosome. (14) The recognition of tmRNA internal ORF codon by the aminoacyl tRNA induces GTP hydrolysis. The aa-tRNA^*aa*^ is accommodated in the A-site. EF-Tu•GDP and the E-Site deacyl tRNA are released. (15) Peptidyl transfer. The nascent peptide is transferred to the incoming aa-tmRNA^*aa*^ and is elongated by one amino-acid. (16) Ratchet. (17) EF-G•GTP binding. (18) GTP hydrolysis and translocation. (19) EF-G•GDP release. The process is repeated until the tmRNA STOP codon is reached. After the first cycle, like deacyl tRNAs, the TLD and SmpB are released from the E-site. Termination-recycling: (20) The tmRNA STOP codon is reached. (21) RF1 or RF2 recognize the STOP codon and bind to the A-site. (22) The class I release factor triggers P-site tRNA deacylation. The new peptide (if unfolded) or protein (if already folded by the CFM) carrying the tmRNA tag is released. A protease such as ClpXP recognizes the tag and degrades the potentially-hazardous product. (23) Class II release factor binds to the ribosome. (24) GTP hydrolysis induces a ratchet-like movement and rapid dissociation of class I and II release factors and E-site deacyl-tRNA. (25) RRF and EF-G•GTP binding. (26) GTP hydrolysis. RRF acts as a wedge, inducing dissociation and recycling of the large ribosomal subunit. RRF and EF-G•GDP are also released. (27) Deacyl tmRNA-SmpB and tRNA dissociate from the small ribosomal subunit. The 30S can be used for a new round of translation. tmRNA-SmpB is recycle. Abbreviations: Rnase III, endoribonuclease III; Rnase E, endoribonuclease E; Rnase T, exoribonuclease T; Rnase PH, exoribonuclease PH; Rnase P, endoribonuclease P; Y: pseudouridine; m5U, 5-methyluridine; TruB, tRNA pseudouridine synthase II; TrmA, S-adenosyl methionine-dependent rna methyltransferase; tmRNA, transfer-messenger RNA; SmpB, small protein B; tmRNA-SmpB, deacyl transfer-messenger RNA and small protein B binary complex; AlaRS, alanyl-tRNA synthetase; ala-tmRNA-SmpB, alanyl transfer-messenger RNA and small protein B binary complex; EF-Tu, elongation factor thermo unstable; GTP, guanosine-5′-triphosphate; S1, small ribosomal subunit protein 1; t-RNA deacyl transfer RNA; EF-G, elongation factor G; mRNA, non-stop mRNA; Rnase R, exoribonuclease R; aa-tRNA, amino-acyl transfer RNA; CFM, co-translational folding machinery; RF1/2, release factor 1 or 2; ClpXP, a protease complex; RF3, release factor 3; RRF, ribosome recycling factor; 50S, large ribosomal subunit; 30S, small ribosomal subunit.

Protein synthesis, also called translation, allows for an accurate correspondence between the genetic information stored in cells and synthesized polypeptides. In bacteria, when ribosomes reach the 3′-end of “non-stop” messenger RNAs (mRNAs), they become non-productive translation complexes (NTCs). This ribosome stalling is a serious issue for bacterial survival, and rescue systems are needed in order to maintain cell viability. The primary rescue system that permits ribosome release is *trans*-translation, mediated by transfer-messenger RNA (tmRNA) and small protein B (SmpB) (Giudice and Gillet, 2013). In a sophisticated ballet, this “all-in-one” complex uses available translation factors to restore protein synthesis, eject the truncated mRNA from the stalled ribosome, and tag the nascent protein for immediate destruction by proteases (Figure 1).

tmRNA was first discussed in the literature in 1979, when Ray and Apirion described a new stable “10S RNA” molecule in *Escherichia coli* (Ray and Apirion, 1979). Although the gene encoding for this small stable RNA was described more than 10 years later in the *E. coli* chromosome (Oh et al., 1990), it was not until 1996 that its physiological role was finally understood. At that time, Keiler et al. described tmRNA's peptide tagging activity in the degradation of proteins synthesized from damaged mRNA (Keiler et al., 1996). Who would have expected then that this new RNA, which surprisingly contains both a tRNA-like structure (Komine et al., 1994) and an mRNA open reading frame (ORF), would occupy the time and efforts of so many laboratories? This was however just the beginning of a long journey that led to the naming of the process as “*trans*-translation.” *Trans*-translation is carried out by hybrid transfer-messenger RNA (tmRNA, formerly SsrA, 10S or 10Sa RNA), in company with small protein B (SmpB), a unique RNA-binding protein essential to the process (Karzai et al., 1999). Since then, more than 450 articles have been published in the field, yielding an accurate description of this finely-tuned process at the molecular level (Moore and Sauer, 2007; Giudice and Gillet, 2013). Thus, almost 35 years after its discovery, tmRNA finally came of age, and it was designated molecule of the month by the RCSB Protein Data Bank in January 2013 (http://www.rcsb.org). This article, part of the first special issue dedicated to *trans*-translation and alternative pathways, aims at elucidating the mechanistics of the process at the molecular level, with particular attention paid to how structural data has helped explain the manner in which the tmRNA-SmpB complex targets and frees stalled ribosomes in all bacteria types.

## Transfer-messenger RNA (tmRNA) structures

**Figure 2:**
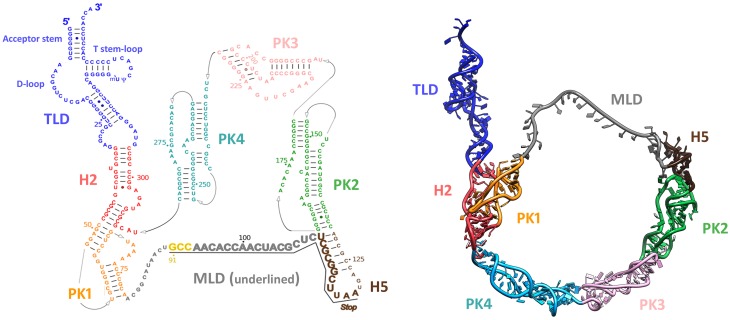
**Structure of tmRNA**. Left: Diagram of the secondary structure of *Thermus thermophilus* tmRNA. Watson-Crick base pairs are connected by lines, and GU pairs are represented by dots. Domains are highlighted with colors: tRNA-like domain (TLD) is blue; helix 2 (H2) is red; pseudoknot 1 (PK1) is orange; the single strand portion carrying the messenger-like domain (MLD) is gray; helix 5 (H5) is brown, pseudoknot 2 (PK2) is green; pseudoknot 3 (PK3) is pink; and pseudoknot 4 (PK4) is teal. The codons are underlined and shown in a larger font. The resume codon is yellow and the stop codon is indicated. Right: 3D molecular model of tmRNA (PDB entry: 3IYQ chain A). The model was constructed using homology modeling on each independent domain, followed by flexible fitting into the cryo-EM density map of the translocated step (EMDB entry: EMD-5189). The same color codes are used as in the left panel.

tmRNA is a remarkable chimeric molecule with both transfer and messenger RNA activities. It ranges from 230 to 400 nucleotides in length. Its modular and highly-structured architecture includes a tRNA-like domain (TLD), a huge ring made of pseudoknots (PKs), a long and disrupted helix H2 connecting the TLD to the PKs, and a short mRNA-like domain (MLD) made of a single strand portion as well as a conserved helix H5 carrying a termination codon (Figure 2).

### The tRNA-like domain (TLD)

The interactions between the 5′- and 3′-ends of the mature tmRNA molecule form an acceptor stem. Like regular tRNA, this stem is extended by a 3′-terminal cytidine-cytidine-adenosine trinucleotide (CCA), but it can only be aminoacylated with alanine (Komine et al., 1994; Ushida et al., 1994). The domain also has a tRNA-like T stem-loop, but its D-loop is reduced and has no stem. Specific interactions between these two loops are required for SmpB binding and function (Barends et al., 2002). The T-loop is also subject to post-transcriptional modifications, and contains two modified nucleosides: 5-methyluridine and pseudouridine (Felden et al., 1998; Ranaei-Siadat et al., 2013).

### The mRNA-like domain (MLD)

The MLD contains a short internal ORF which includes a stop codon and encodes for a tag immediately recognizable by proteases. This conserved tag is usually made up of 10 residues (AANDENYALAA in *E. coli*, with the first A carried by tmRNA) (Moore and Sauer, 2007), although it can contain 8–35 residues. Contrary to canonical mRNA, it does not carry any start sites positioned upstream to the AUG initiation codon, such as a Shine-Dalgarno sequence. Instead, the resume codon is in most cases an alanine and sometimes a glycine codon. The five nucleotides immediately upstream of this first codon appear to direct frame selection (Watts et al., 2009). Once released, the tagged protein is degraded by various enzymes. In the *E. coli* cytoplasm, this is done mainly by ClpXP, ClpAP, and FtsH proteases (Karzai et al., 2000), while Tsp, an energy-independent protease, performs the same task within the periplasm. Within the usual tagging sequence AANDENYALAA, ClpX binds the C-terminal residues LAA, while ClpA binds the C-ter residues ALA and makes additional contacts with the N-terminal residues AA (Janssen and Hayes, 2012). ClpXP performs the majority of degradation, with FtsH degrading just a small subset of proteins that are present in the inner membrane.

### The ring of pseudoknots

Generally, tmRNA has four pseudoknots (PKs). PK1 is upstream from the MLD and PK2-PK4 are located downstream. tmRNA tagging requires PK1 but not the others, and the functioning of tmRNA is not seriously affected by the replacement or interchange of any of the other pseudoknots in *E. coli* (Nameki et al., 2000). However, recent research has shown that *in vitro* or *in vivo* substitution of a small and stable RNA hairpin for PK1 still permits tmRNA tagging (Tanner et al., 2006). This suggests that instead of having a direct role in ribosome binding, PK1 must help stabilize the region enclosed by the TLD and the MLD, and prevent tmRNA misfolding (Tanner et al., 2006; Wower et al., 2009). The primary role of PK2, PK3, and PK4 is the folding and maturation of tmRNA rather than its *trans*-translational activity (Wower et al., 2004). Accordingly, in certain classes of active tmRNAs (“two-piece tmRNAs”), a dramatic reduction in pseudoknot number is observed without a decrease in tagging efficiency (Gaudin et al., 2002). These two-piece tmRNAs have been observed in alpha-proteobacteria, cyanobacteria, and some beta-proteobacteria lineages, and result from gene circular permutation that split them into two molecules (Keiler et al., 2000; Sharkady and Williams, 2004). They have a TLD and an MLD, but fewer pseudoknots than their one-piece ancestors (Gaudin et al., 2002).

## Small protein B (SmpB) structure

**Figure 3:**
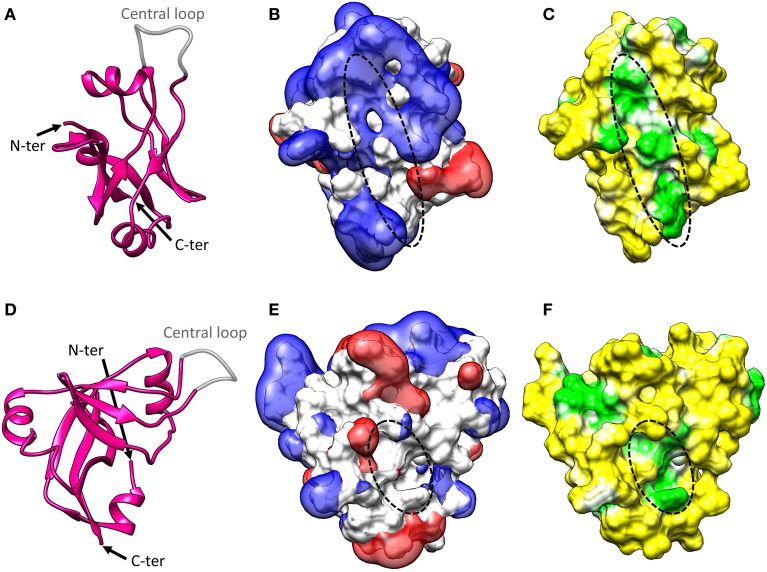
**SmpB structure. (A)** Cartoon representation of the crystal structure of *Thermus thermophilus* SmpB (PDB entry: 1WJX). The N-terminal end, C-terminal end, and central loop are indicated. SmpB adopts an oligonucleotide-binding fold (OB fold) with a central β-barrel and three flanking α-helices. The C-terminal tail is unstructured in solution but folds into a fourth α-helix once SmpB is inserted into the ribosome. The central loop is disordered in the crystal, suggesting that it must be flexible when the protein is alone. **(B)** Electrostatic potential of SmpB. Two isocontours at −1 V (red) and +1 V (blue) are represented with the solvent-accessible surface of SmpB (white). The potential was calculated using the APBS program (Baker et al., 2001) with CHARMM force field parameters and an ionic strength of 50 mM. The primary tmRNA binding site interacting with the TLD (indicated with a dotted line) is surrounded by a strong electropositive field. **(C)** Molecular hydrophobicity potential projected on the solvent-accessible surface of SmpB. The potential was computed with the Platinum server using Ghose force field parameters (Pyrkov et al., 2009). The hydrophobicity scale is green-white-yellow, with yellow representing the most hydrophilic regions and green the most hydrophobic. The primary tmRNA binding site interacting with the TLD (indicated with a dotted line) is formed by a deep hydrophobic patch. **(D–F)** Side views of the information presented in **(A–C)**. Note that in **(E,F)**, a dotted line indicates the secondary tmRNA binding site that interacts with the nucleotides upstream from the resume codon after translocation of tmRNA-SmpB into the ribosomal P-site.

**Figure 4:**
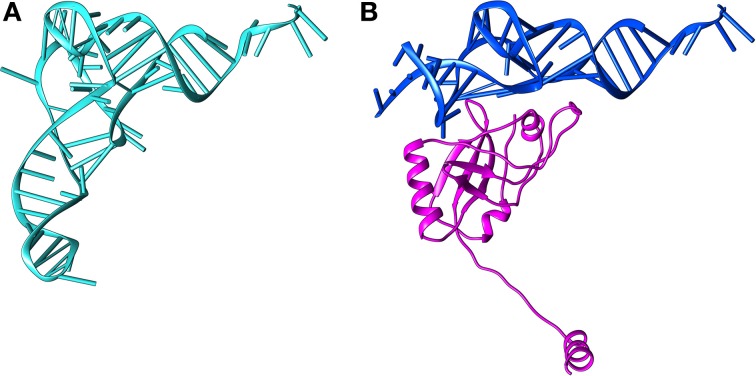
**Structural comparison between tRNA and the tRNA-like domain of tmRNA bound to SmpB. (A)** The structure of tRNA (PDB entry 2WRN). **(B)** The structure of TLD-SmpB (PDB entry 4ABR). The TLD is blue and SmpB is magenta. The TLD resembles the upper part of a tRNA, with SmpB replacing the tRNA anticodon stem-loop.

**Figure 5:**
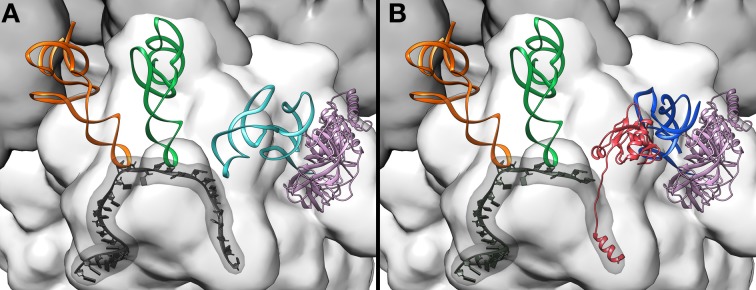
**Comparing ribosomal A-site recognition by canonical tRNA and tmRNA–SmpB. (A)** tRNA pre-accommodated on the ribosome (Schmeing et al., 2009) (PDB entries 2WRN, 2WRO). The large 50S subunit is dark gray the, small 30S subunit is light gray, mRNA is black (the mRNA path is also highlighted), the E-site tRNA is orange, the P-site tRNA is green, EF-Tu is pink, and the incoming tRNA is light blue. **(B)** The TLD- SmpB complex pre-accomodated on the ribosome (Neubauer et al., 2012) (PDB entries 4ABR 4ABS). The C-terminal tail of SmpB folds into an α-helix inserted within the empty mRNA path. The color code is as **(A)**, with TLD in blue and SmpB in red.

**Figure 6:**
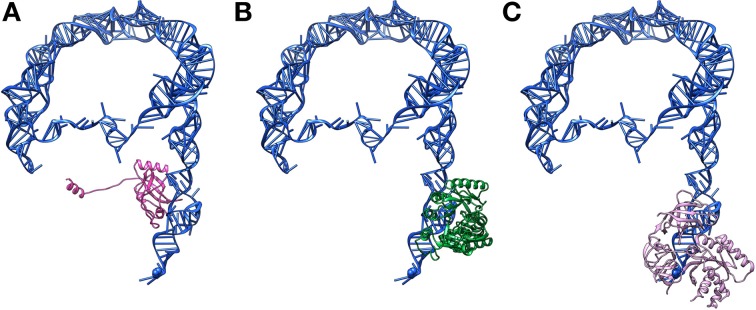
**The tRNA-like domain is the primary binding site for most tmRNA protein partners. (A)** Atomic model of the tmRNA-SmpB complex based on PDB entry 4ABR, and EMDB entry EMD-1312. tmRNA is blue and SmpB is magenta. **(B)** Atomic model of the tmRNA-TrmA complex based on PDB entries 4ABR and 2OB7, and EMDB entry EMD-1312. tmRNA is blue and TrmA is green. **(C)** Atomic model of the tmRNA-EF-Tu complex based on PDB entry 4ABR and EMDB entry EMD-1312. tmRNA is blue and EF-Tu is pink.

SmpB is a small basic protein (160 amino-acids in *E. coli*) essential for *trans*-translation (Karzai et al., 1999). All bacterial genomes contain the *smpb* gene with high primary sequence conservation. Deleting this gene results in the same phenotypes as those observed in cells lacking tmRNA. The first SmpB structure was solved with NMR studies (Dong et al., 2002; Someya et al., 2003). These revealed that the protein adopts an oligonucleotide-binding (OB) fold made up of six antiparallel β-strands arranged in the typical closed β-barrel surrounded by three α-helices. Two conserved RNA-binding domains on opposite sides of the protein are thus exposed (Figure 3). Further X-ray studies have shown that SmpB binds with high specificity to the TLD elbow region, stabilizing the single-stranded D-loop in an extended conformation (Gutmann et al., 2003; Bessho et al., 2007). The binding activity increases the elbow angle to about 120° (as opposed to 90° in canonical tRNAs), a change which electrical and birefringence studies had already suggested (Stagg et al., 2001). This puts SmpB where one usually finds the anticodon and D stems of tRNA, and the tmRNA H2 helix (Figures 4, 5, 6A) mimics a long tRNA variable arm. SmpB also has a C-terminal tail (residues 131–160 in *E. coli*) which, although always unstructured in solution, forms an α-helix in the ribosome (Figures 4B, 5B, 6A). This tail is essential for tmRNA tagging (Dong et al., 2002; Someya et al., 2003; Jacob et al., 2005; Sundermeier et al., 2005; Shimizu and Ueda, 2006; Gillet et al., 2007) and was recently shown to bind to the 30S A-site (Neubauer et al., 2012) as previously predicted (Kaur et al., 2006; Nonin-Lecomte et al., 2009; Kurita et al., 2010). Notably, the most conserved residues are not in the helix but just upstream (Miller et al., 2011). The conservation of this unstructured portion may be necessary to maintain flexibility and ensure the correct positioning of the helix. Acting together with the residues forming the second RNA-binding domain site, it may also play a role in the selection of the correct codon. It has to be noted that it is only the body of SmpB that is responsible for its binding affinity in the decoding center, while the entering of the C-terminal tail into the mRNA channel would account for the release of EF-Tu and the proper accommodation of tmRNA-SmpB in the decoding center (Miller and Buskirk, 2014).

## Other tmRNA partners

SmpB is not the only RNA-binding protein needed for tmRNA's *trans*-translational activities. Other necessary proteins include: processing enzymes; enzymes catalyzing post-transcriptional modifications; alanyl tRNA synthetase (AlaRS); EF-Tu; S1; and RNase R (Saguy et al., 2005). All of the known tmRNA partners bind to its TLD (Figure 6), with the notable exception of S1 which can interact with the PK ring.

### Processing enzymes

As with canonical tRNA genes, the tmRNA-encoding *ssrA* gene also encodes for a primary transcript that needs to be processed before yielding to the fully-mature molecule (Keiler et al., 2000). This precursor is 457-nucleotides long in *E. coli*, and is processed at the 5′-terminus by the endonuclease RNAse P (Komine et al., 1994). The 3′-terminus is cleaved by the endonucleases RNAse III or E, then trimmed by exonucleases RNAse T and/or PH (Li et al., 1998).The result is a 363 nt-long mature *E. coli* tmRNA with a conserved 3′-terminal CCA trinucleotide (Lin-Chao et al., 1999).

### Enzymes catalyzing post-transcriptional modifications

As in regular tRNA, post-transcriptional modifications occur in tmRNA. In particular, two modified nucleosides, 5-methyluridine (m5U) and pseudouridine (Ψ), have been identified in the T-loop of the molecule's TLD (Felden et al., 1998). In *E. coli*, three methyltransferases can catalyze the C5-methylation of uridine: TrmA (formerly RumT); RlmD; and RlmC. However, it has been recently demonstrated that only TrmA is responsible for this process in tmRNA (see Figure 6B for a model of the interaction) (Ranaei-Siadat et al., 2013). Since this enzyme is absent in Gram-positive bacteria, a methylene-tetrahydrofolate dependent enzyme, TrmFO, probably takes over this responsibility in such bacteria (Ranaei-Siadat et al., 2013). Pseudouridylation, meanwhile, is probably performed by the tRNA Ψ55 synthase (“TruB”), which is responsible for the same modification in the tRNA T-loop (Felden et al., 1998; Ranaei-Siadat et al., 2013).

### Alanyl-tRNA synthetase (AlaRS)

The conserved 3′-terminal tail of tmRNA is always charged with an alanine by alanyl-tRNA synthetase (AlaRS), a class II tRNA synthetase that catalyzes the esterification of alanine to tRNA^Ala^. The presence of the G3 U70 wobble base pair (found in the acceptor stem of all tRNA^Ala^ isoacceptors) and of an adenosine at the discriminator position adjacent to the 3′-terminal CCA are the keys to specific recognition of tRNA^Ala^ by AlaRS (Hou and Schimmel, 1988). Interestingly, this same wobble base pair is conserved in all *ssrA* sequences, which makes AlaRS the only amino-acid synthetase working on tmRNA *in vivo*. Although the structural details of the AlaRS-tmRNA interaction have not yet been elucidated, it is possible that one of the SmpB loops is involved in the interaction with alanyl-tRNA synthetase, which would explain why the protein encourages tmRNA alanylation (Bessho et al., 2007).

### Elongation factor EF-Tu

EF-Tu accounts for up to 5% of the total cellular protein, making it the most abundant protein in the bacterial cell. It forms a ternary complex with aminoacyl-tRNA (aa-tRNA) and GTP, bringing aa-tRNA to the ribosome (Kavaliauskas et al., 2012). The same goes for *trans*-translation, where EF-Tu•GTP interacts with tmRNA-SmpB to form a quaternary complex and initiate the rescue of the stalled ribosomes (Figure 6C). A *trans*-translating ribosome in its pre-accommodated stage has a very similar structure to that of the equivalent EF-Tu-aa-tRNA complex, including the specific 3′-CCA end and acceptor arm conformations along with a T-arm that interacts with EF-Tu. However, EF-Tu and SmpB do not interact (Neubauer et al., 2012) Surprisingly, EF-Tu•GDP can also bind to charged or deacylated tmRNA (Zvereva et al., 2001; Stepanov and Nyborg, 2003). In cases such as these, EF-Tu also interacts with regions outside the TLD, and this unexpected activity may protect tmRNA from degradation. Last but not least, it was recently shown that release of EF-Tu from the tmRNA-SmpB complex on the ribosome may occur prior to GTP hydrolysis (Miller and Buskirk, 2014).

### Ribosomal protein S1

S1 is the *rpsA* gene product and the longest and largest of the ribosomal proteins. In Gram-negative bacteria, its weak association with the 30S small subunit makes it a key mRNA-binding protein, as it facilitates ribosomal recognition of most mRNAs during translation initiation (Sorensen et al., 1998; Hajnsdorf and Boni, 2012). The protein is made of six homologous domains (“S1” domains) that characterize the OB-fold family of RNA-binding proteins (Bycroft et al., 1997). S1's N-terminal domain binds to the ribosome, leaving its elongated C-terminal RNA-binding domain protruding into solution (Subramanian, 1983). Since S1 binds to tmRNA 600 times better than to tRNA, S1 must play an important role in *trans*-translation, forming complexes with free tmRNA and then promoting ribosomal binding (Wower et al., 2000). While the TLD remains unaffected, significant conformational changes have been observed in tmRNA pseudoknots upon S1 binding. This suggests that S1 binds tmRNA by contacting the PK ring, interacting most strongly with PK2 (Bordeau and Felden, 2002). Interesting clues to the role played by S1 during *trans*-translation come from cryo-electron microscopic (cryo-EM) data showing how tmRNA binds to the ribosome in a pre-accommodated step (Valle et al., 2003; Gillet et al., 2007). Without S1, the tmRNA ribosome complex displays an extra density which corresponds to the MLD. This suggests that S1 is involved in the unwinding of the MLD outside the ribosome before initiation of *trans*-translation. Thus even before tmRNA-SmpB binds to the ribosome, S1 might enter the PK ring (which has an inner diameter of about 80Å), facilitating the access to the internal ORF (Bordeau and Felden, 2002). Then once the tmRNA binds to the stalled ribosome, S1 would be released, and the ORF placed in the decoding site (Gillet et al., 2007). Recent data on the Gram-positive *Actinobacteria* group confirm the indispensability of S1 for *trans*-translation. Indeed, the first-line anti-tuberculosis drug pyrazinamide inhibits *trans*-translation by transforming into pyrazinoic acid, a molecule which binds to S1 (Shi et al., 2011).

### Exoribonuclease R

**Figure 7:**
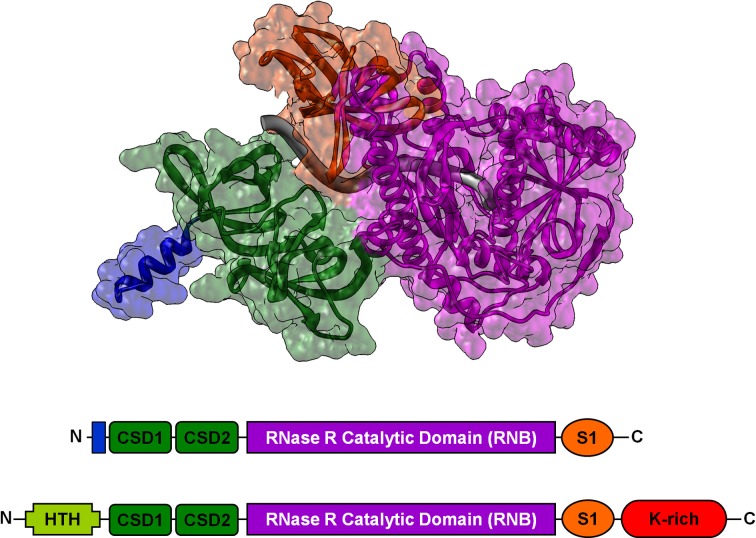
**Comparison of 2D and 3D structures of Rnase II and Rnase R. Top:** Cartoon and surface representation of the crystal structure of *RNase II* D209N mutant bound to an RNA fragment (Frazao et al., 2006) (PDB entry 2IX1). The N-terminal domain is blue, cold-shock domains are green, the catalytic domain is magenta, the S1 domain is orange, and the RNA fragment is gray. **Bottom**: Schematic representation of the RNase R and RNase II domain architectures. RNase R and RNase II are very similar, with two cold-shock domains, a large central catalytic domain, and the S1 domain. However RNase R has two additional domains: an N-terminal putative helix–turn–helix (HTH) domain, and a C-terminal lysine-rich (K-rich) domain crucial for its non-stop mRNA degradation activity.

Exoribonuclease R (RNase R) is a member of the RNase II superfamily, a group of enzymes that degrade RNA through hydrolysis, moving progressively in a sequence-independent manner in the 3–5′ direction. In *E. coli*, RNase R is a ubiquitous *rnr*-encoded 92 kDa protein. RNase R has helicase activity, and helps degrade structured RNAs, including small, ribosomal, and messenger RNAs (Cheng and Deutscher, 2002; Matos et al., 2011). During *trans*-translation, to avoid being recruited over and over in a feedback loop of translation and *trans*-translation, problematic mRNA transcripts must be degraded quickly. Thanks to its unique K-rich domain (Figure 7), RNase R is recruited to stalled ribosomes to degrade the defective mRNAs in a *trans*-translation-dependent manner (Richards et al., 2006; Ge et al., 2010). Strikingly, tmRNA-SmpB has several distinct roles in regulating the stability and action of RNase R. tmRNA-SmpB binding to the C-terminal K-rich domain of RNase R is required for the enzyme's recruitment to stalled ribosomes (Ge et al., 2010). RNase R acetylation, observed mostly during the exponential phase, also promotes tmRNA-SmpB binding to the C-terminal domain. However, this in turn stimulates the degradation of RNase R by HslUV and Lon proteases (Liang and Deutscher, 2012, 2013). In *Caulobacter crescentus*, tmRNA cycle-regulated degradation is ensured by RNase R, but its action is regulated by SmpB (Hong et al., 2005). Finally, it was recently shown in *Streptococcus pneumoniae* that SmpB and RNase R are both cross-regulated and co-transcripted (Moreira et al., 2012). *Trans*-translation and RNase R are thus obviously tightly interdependent.

## Structural aspects of *trans*-translation

**Figure 8:**
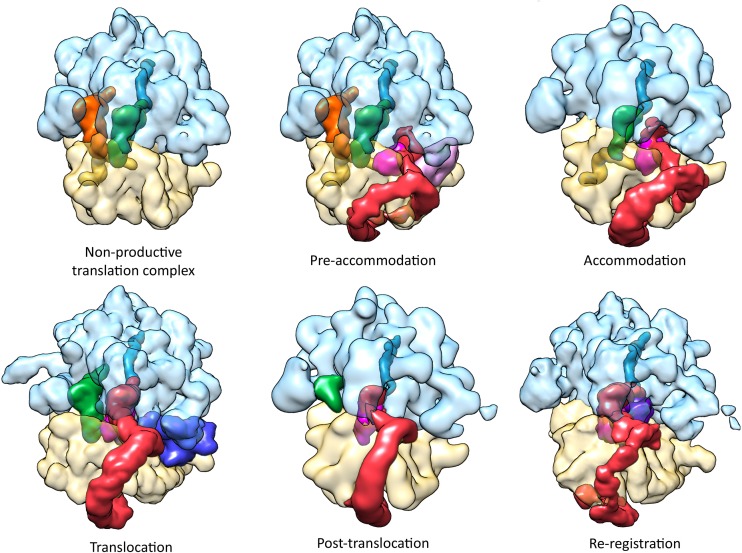
**Cryo-EM maps of the currently-solved *trans*-translation functional complexes**. Non-productive translation complex (NTC): the ribosome stalls at the 3′-end of the mRNA, the A-site lacks a complete codon. Pre-accommodation: The alanyl-tmRNA•SmpB•EF-Tu•GTP quaternary complex enters the vacant A-site of a non-stop stalled ribosome (EMDB entry: EMD-1312) (Valle et al., 2003; Kaur et al., 2006). Accommodation: EF-Tu dissociates after GTP hydrolysis, allowing tmRNA-SmpB to occupy the A-site (EMDB entry: EMD-5188) (Weis et al., 2010a). Translocation: EF-G•GTP catalyzes the translocation of tmRNA-SmpB to the P-site. The ribosome is in a ratcheted state and a unique swivel of the head is observed (EMDB entry: EMD-5386) (Ramrath et al., 2012). Post-translocation: After dissociation of EF-G•GDP, the subunits return to normal positioning, and the resume codon of tmRNA is correctly placed into the A-site (EMDB entry: EMD-5189) (Weis et al., 2010a). Re-registration: Translation switches on the tmRNA internal ORF, and a new aminoacyl-tRNA binds to the resume codon (EMDB entry: EMD-5234) (Fu et al., 2010). Color code: the large 50S subunit is light blue; the small 30S subunit is pale yellow; the truncated mRNA is yellow; the nascent polypeptide is teal; the tRNA initially occupying the E-site is orange; the tRNA initially occupying the P-site is green; the tmRNA is red; the SmpB is magenta; the EF-Tu is light pink; the EF-G is blue; and the A site-tRNA is purple.

Most of the initial steps of *trans*-translation have been made clear by cryo-EM studies. These studies resulted in several structures of the pre-accommodation, accommodation, and first translocation steps of tmRNA-SmpB on the ribosome (Figure 8). Crystal and NMR structures of isolated SmpB and TLD-SmpB also paved the way for an accurate positioning of tmRNA and partners into the cryo-EM maps. More recently, the crystal structure of a tmRNA fragment with SmpB and EF-Tu bound to the ribosome has greatly increased the understanding of how the tmRNA-SmpB complex interacts with a stalled ribosome without interfering in normal translation (Figure 1).

### Pre-accommodation

**Figure 9:**
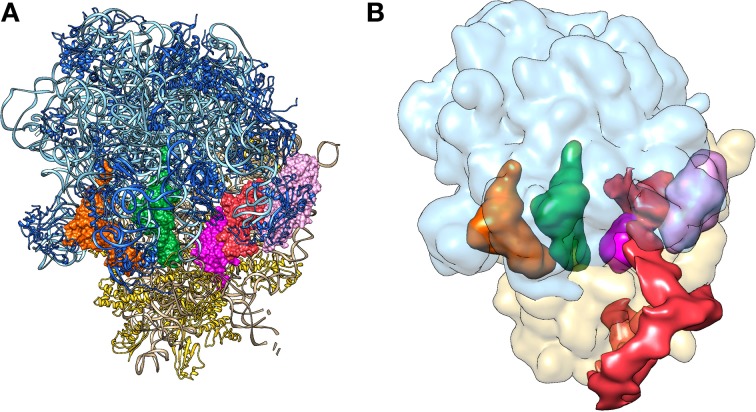
**The pre-accommodation step of *trans*-translation as solved by X-ray diffraction and to cryo-electron microscopy. (A)** Crystal structure of the alanyl-TLD-SmpB-EF-Tu•GDP quaternary complex bound to a stalled ribosome (Neubauer et al., 2012) (PDB entries 4ABR 4ABS). **(B)** Cryo-EM map of the alanyl-tmRNA-SmpB-EF-Tu•GTP quaternary complex bound to a stalled ribosome (Valle et al., 2003) (EMDB entry: EMD-1312). Color code: tmRNA or TLD is red; SmpB is magenta; the small 30S subunit is yellow; the large 50S subunit is light blue; the E-Site tRNA is orange; the P-site tRNA is green; and the EF-Tu is pink.

The first structure of the tmRNA-SmpB-EF-Tu•GDP pre-accommodation step was obtained by cryo-EM (Valle et al., 2003) and was helpful in understanding how the ribosome and the huge tmRNA molecule interact. The images demonstrated that the TLD is associated with EF-Tu, and that it is guided to the ribosome in the same manner as an aminoacyl tRNA (Figure 8). Combining the crystal structures of the TLD-SmpB complex in solution (Gutmann et al., 2003; Bessho et al., 2007) and chemical probing experiments (Kurita et al., 2007), it was suggested that SmpB must mimic tRNA's codon-anticodon pairing and D stem-loop, with the TLD playing the role of tRNA's upper half (Figure 4). There has been some controversy about the number of SmpB molecules involved in *trans*-translation (Felden and Gillet, 2011). However, in keeping with the most recent crystal and cryo-EM studies of pre- and post-accommodated states, the agreed upon model has a 1:1 SmpB:tmRNA molar ratio. The cryo-EM maps were also helpful in understanding how the ribosome and the huge tmRNA molecule interact. In fact, the helix H2 mimics a long tRNA variable arm, leaning along the 30S subunit and pointing out of the ribosome toward the beak. The other domains (PK1, the MLD, H5, PK2, PK3, and PK4) wrap around the 30S beak like a ribbon (Figures 8, 9). The recent crystal structure of TLD-SmpB-EF-Tu on the ribosome has revealed even more details about how the tmRNA-SmpB complex identifies stalled ribosomes (Neubauer et al., 2012). Acting in every way as a tRNA molecule, after binding to the ribosome the TLD-SmpB complex shows no major distortions (Figure 9). A *trans*-translating ribosome in its pre-accommodated stage has an overall conformation closely resembling that of an equivalent complex of EF-Tu with aminoacylated tRNA. Similarities include the conformations of the 3′-CCA end, the acceptor arm, and the T-arm portions (Figure 5). To bring the shoulder domain of the 30S subunit (containing the key residue G530) closer to its 3′ major domain (containing the decoding residues A1492 and A1493), SmpB interacts with the 30S subunit. In doing so, SmpB tricks it into adopting a “closed” conformation, as if in the presence of a cognate codon-anticodon, with the shoulder and head domains rotated toward the subunit's center (Ogle et al., 2002). In the unoccupied mRNA pathway downstream of the decoding center, the SmpB C-terminal tail simultaneously folds into an α-helix (Figure 5B). This allows the protein to undergo specific interactions with regions only accessible in the absence of mRNA, thus stabilizing SmpB and permitting an accurate identification of the vacant A-site. The pre-accommodation structure therefore explains the functional relevance of the SmpB C-terminal tail in tmRNA tagging. Interestingly, the same strategy is adopted by ArfB, a recently-discovered alternative rescue factor. ArfB possesses a structure similar to the catalytic domain of class I release factors. It also has a helical C-terminal tail which binds in the mRNA entry channel of the small subunit, allowing discrimination between active and stalled ribosomes (Gagnon et al., 2012).

### Accommodation

After GTP hydrolysis, EF-TU•GDP is released and the tmRNA-SmpB complex accommodates into the A-site. The TLD contacts with the large ribosomal subunit look like those of an accommodated canonical tRNA (Cheng et al., 2010; Fu et al., 2010; Weis et al., 2010a,b). In this step, the D-loop interacts with helix H38 and the acceptor branch guides the CCA 3′-end into the peptidyl transfer center. The TLD swings into the A-site, and SmpB follows, rotating by about 30° while still mimicking an anticodon stem-loop (Figure 8). Helix H2 therefore realigns itself toward the large subunit, interacting with protein L11. The PK ring does not undergo large movements and stays wrapped around the beak of 30S.

### Translocation

After transpeptidation, nascent peptides are elongated by one alanine, and there is a spontaneous rotation of the 30S subunit in an anticlockwise direction from the 50S. This ratchet-like motion brings tmRNA and tRNA into hybrid states of binding (A/P and P/E, respectively). During this translocation reaction, EF-G binds to SmpB as it does to tRNA, but it triggers a unique 12° tilt of the 30S head (Ramrath et al., 2012). The tmRNA-SmpB complex is in a hybrid state, with the TLD bound to the 50S P-site, and SmpB still pointing toward the A-site. The opening of the inter-subunit B1a bridge (or “A-site finger”) during the ratchet movement allows helix H2 to go through (Weis et al., 2010a). Thus the A-site finger, whose mutations are known to alter tmRNA function (Crandall et al., 2010), interacts with PK1. After the head moves, the pseudoknot ring rotates, allowing H5/PK2 to come into contact with proteins S2 and S3 at the 30S subunit's surface at the same time as the tmRNA internal ORF extends into the mRNA path (Figure 8). After EF-G•GDP disassociates, the subunits return to their usual positions, and the tmRNA resume codon is placed into the A-site (Weis et al., 2010a). Translation then re-registers on the tmRNA internal ORF, and a new aminoacyl-tRNA complex binds to the resume codon. During these “post-translocation” and “re-registration” steps, SmpB remains bound to the tmRNA (Shpanchenko et al., 2005) and the TLD-SmpB takes up the same space in the P-site as a regular tRNA would. Helix H2 is inserted tightly between the two ribosomal subunits, forming several contacts with both. The PK ring's conformation and orientation mostly remains the same, with H5 and PK2 staying at the 30S subunit's surface. At this stage, since helix H5 is still present, the internal ORF is only partially unfolded. However the distance separating PK1 and H5 increases, which suggests that the single strand connecting the two domains must be fully extended during its insertion into the mRNA path. While this single-strand section of the tmRNA cannot be directly seen in the cryo-EM structure, the ribosomal environment is sufficiently restrained so that it can be modeled precisely. Molecular dynamics flexible fitting showed that the upstream region of the tmRNA resume codon interacts with the C-terminal tail of SmpB and with the hydrophobic pocket at the bottom of the protein (Fu et al., 2010; Weis et al., 2010a). These interactions place the resume codon directly in the 30S decoding center. This explains the essential role in frame selection of the five nucleotides upstream from the tmRNA resume codon (Williams et al., 1999; Lee et al., 2001; Miller et al., 2008), of the SmpB C-terminal tail (Miller et al., 2011), and of four specific residues on the SmpB surface (Watts et al., 2009). Comparing the accommodation and post-translocation electron density maps confirms the release of truncated mRNA during the translocation of tmRNA to the P-site (Weis et al., 2010a).

### Moving forward into the ribosome

The remaining steps have not yet been observed, but a *trans*-translation model can be proposed by comparing it to canonical translation. It is certain that tRNA and the tmRNA-SmpB complex are translocated into the P- and E-sites, respectively, and that this process is repeated until reaching the tmRNA stop codon. Along the way, the TLD and H2 of SmpB and tmRNA are rapidly released. The messenger part of tmRNA is extended, leading to a deconstruction of helix H5 (Wower et al., 2005; Bugaeva et al., 2009). The tmRNA-SmpB complex stays near the ribosome, as the internal ORF is on the mRNA pathway. When the stop codon is reached, a class I release factor (either RF1 or RF2) binds to the A-site, inducing hydrolysis of the nascent peptide from the P-site tRNA. The protein is then released from the ribosome and immediately targeted by proteases because of its tagged C-terminal tail. At the end of the termination/recycling step, the deacyl-tRNA, deacyl-tmRNA-SmpB, and ribosomal small subunits separate, and the tmRNA-SmpB complex is recycled (Figure 1).

## Concluding remarks

tmRNA-SmpB structures inside and out of the bacterial ribosome have provided a framework for understanding how bacteria cope with stalled protein synthesis. In combination with genetic and biochemical studies, such data have yielded a clear model of *trans*-translation at the molecular level (Figure 1). Finally, several decades after the discovery of this process, technological and therapeutic developments should be possible. The recent discovery that *trans*-translation can be a target for several antibiotics confirms its high therapeutic potential. We hope that researchers will now be able to exploit structural insights into the *trans*-translating ribosome, leading to new antibiotics that target the bacterial ribosome at the quality control level (Ramadoss et al., 2013).

### Conflict of interest statement

The authors declare that the research was conducted in the absence of any commercial or financial relationships that could be construed as a potential conflict of interest.
